# Genome-wide surveys reveal polarity and cytoskeletal regulators mediate LKB1-associated germline stem cell quiescence

**DOI:** 10.1186/s12864-018-4847-y

**Published:** 2018-06-15

**Authors:** Pratik Kadekar, Rita Chaouni, Emily Clark, Anna Kazanets, Richard Roy

**Affiliations:** Department of Biology, McGill University, 1205 avenue Docteur Penfield, Montreal, Quebec H3A 1B1 Canada

**Keywords:** PAR-4, AMPK, Actin cytoskeleton, Peutz-Jeghers Syndrome, Dauer, Germ line, *C. elegans*

## Abstract

**Background:**

*Caenorhabditis elegans* can endure long periods of environmental stress by altering their development to execute a quiescent state called “dauer”. Previous work has implicated LKB1 - the causative gene in the autosomal dominant, cancer pre-disposing disease called Peutz-Jeghers Syndrome (PJS), and its downstream target AMPK, in the establishment of germline stem cell (GSC) quiescence during the dauer stage. Loss of function mutations in both LKB1/*par-4* and AMPK/*aak(0)* result in untimely GSC proliferation during the onset of the dauer stage, although the molecular mechanism through which these factors regulate quiescence remains unclear. Curiously, the hyperplasia observed in *par-4* mutants is more severe than AMPK-compromised dauer larvae, suggesting that *par-4* has alternative downstream targets in addition to AMPK to regulate germline quiescence.

**Results:**

We conducted three genome-wide RNAi screens to identify potential downstream targets of the protein kinases PAR-4 and AMPK that mediate dauer-dependent GSC quiescence. First, we screened to identify genes that phenocopy the *par-4*-dependent hyperplasia when compromised by RNAi. Two additional RNAi screens were performed to identify genes that suppressed the germline hyperplasia in *par-4* and *aak(0)* dauer larvae, respectively. Interestingly, a subset of the candidates we identified are involved in the regulation of cell polarity and cytoskeletal function downstream of *par-4*, in an AMPK-independent manner. Moreover, we show that *par-4* temporally regulates actin cytoskeletal organization within the dauer germ line at the rachis-adjacent membrane, in an AMPK-independent manner.

**Conclusion:**

Our data suggest that the regulation of the cytoskeleton and cell polarity may contribute significantly to the tumour suppressor function of LKB1*/par-4*.

**Electronic supplementary material:**

The online version of this article (10.1186/s12864-018-4847-y) contains supplementary material, which is available to authorized users.

## Background

One of the defining features of any multicellular organism is their capacity to organize essentially identical cellular units into multiple individual cell types with distinct functional properties. These cells will then sort themselves to later give rise to the diverse tissues and organs that will function in a coordinated manner to support growth and reproduction. To achieve this degree of functional complexity, the contributing cell units must respond to regulatory cues that convey information that dictates such functional distinctions. Spatial/positional and temporal cues are critical throughout development as cells must know precisely when they may need to respond to a given signal and also where they are oriented among their cellular neighbours with respect to the overall body plan.

Tissues themselves must also be polarized to provide structural integrity that is unique to their function, but also for the correct partitioning of cellular constituents including intracellular proteins, organelles and cytoskeletal components [1]. This permits cells to sense and respond to spatiotemporal signals from adjacent cells and/or the surrounding microenvironment. Consistent with this important role, loss of cell polarity often causes cells to become unresponsive to growth inhibitory signals, allowing cells to circumvent differentiation, senescence, and/or apoptosis [2].

Recently, regulators of cell polarity have also been found to converge on signalling pathways that control cell growth and proliferation and energy metabolism [3, 4]. It is therefore not surprising that many tumours possess abnormalities in tissue architecture that are often associated with, or caused by, the inappropriate expression of key cell polarity regulators. Consistent with this, mutations in the polarity-regulating protein kinase LKB1/STK11 have been identified in individuals with Peutz-Jeghers Syndrome (PJS), an autosomal dominant disease that predisposes patients to various types of cancer [5, 6].

LKB1 is a serine/threonine kinase that phosphorylates 13 other protein kinase substrates in the cell, enabling them to subsequently phosphorylate their respective downstream targets more efficiently [7]. The misregulation of these LKB1 targets may explain how loss of this kinase results in aberrant tissue growth and tumour formation, as they are involved in diverse cellular processes ranging from metabolic control to regulation of epithelial and neuronal polarity.

Among the known LKB1 targets, the activation of AMP-activated protein kinase (AMPK) is perhaps the best characterized, where LKB1 activation of AMPK was found to modulate the TOR pathway to coordinate growth with the metabolic status of the cell [8, 9]. Curiously, PJS shares many clinical features with other hamartomatous syndromes that are presumed to be associated with abnormally increased TOR signalling. These include Tuberous Sclerosis, Cowden’s Disease, and Juvenile Polyposis; all of which share a significantly increased frequency of rare benign hamartomas. AMPK has been shown to phosphorylate Tuberous Sclerosis 2 (TSC2), a GAP protein that blocks TOR activation, and Raptor, an activator of the mTOR pathway that is blocked during periods of starvation [10, 11]. It is therefore compelling to conclude that mutations in LKB1 disrupt this coordination of nutrient/energy availability and growth by compromising AMPK activation, thus leaving TOR signalling unchecked.

However, PJS is an autosomal dominant disease and many patients with PJS retain one wild type copy of LKB1, which is sufficient to activate AMPK [12]. Moreover, no mutation has yet been identified in AMPK in any PJS patient to date, suggesting that LKB1 may act independently of AMPK, and potentially TOR signalling, to suppress tumour growth [13]. Furthermore, data obtained from studies in mice suggest that the tumour suppressor function of LKB1 is not solely TOR-dependent [14]. Moreover, although hyperactivation of TOR signalling has been associated with PJS, inhibition of TOR using rapamycin in LKB1 heterozygous mice indeed reduces polyp numbers in the gut, but does not prevent polyp formation altogether [14]. Therefore, the hyperactivation of TOR that occurs in LKB1+/− individuals may exacerbate the defects in PJS, but it is unlikely to be the unique underlying cause of the disease. This suggests that LKB1 must work through additional TOR-independent targets that contribute to its tumour suppressor function.

We previously showed that both LKB1 and AMPK regulate germline stem cell quiescence in *C. elegans* during periods of energy stress. When either of these genes, or the tumour suppressor PTEN, is impaired, the germline stem cells (GSCs) proliferate when they should normally arrest [15]. Although mutations in either LKB1 or AMPK cause hyperplasia, LKB1 mutations always result in a greater degree of hyperplasia than null mutations that disrupt all AMPK signalling, suggesting that other genes that act downstream of LKB1, and independent of AMPK, must be phosphorylated in order to elicit both cell cycle and developmental quiescence [15].

In *C. elegans,* the defects associated with LKB1 or AMPK disruption are most obvious in the gonads of animals subjected to energy stress. The *C. elegans* germ line develops from two cells that are born during embryogenesis and remain quiescent until the L1 stage. The two cells are referred to as the primordial germ cells Z2 and Z3, which will divide continuously during development in replete conditions to generate all the germ cells that will constitute the adult germ line. The continuous division of these cells is dependent on signalling between the distal-most germ cells and two somatic gonadal cells called distal tip cells (DTCs) that are located at the distal end of each identical growing gonad arm [16].

The DTCs form a niche for the GSCs and their mitotic divisions are maintained through Notch signalling. The Delta-like ligand LAG-2 is expressed in the DTCs, while the GSCs express the Notch-like receptor, GLP-1 [17]. Active Notch signalling instructs these GSCs to proliferate, while blocking them from executing their alternative meiotic pathway. The ongoing divisions driven by Notch signalling physically displace these dividing cells proximally until they no longer receive the Notch signal from the DTCs, allowing them to execute their alternate meiotic pathway [18].

Under optimal environmental conditions germline proliferation continues uninterrupted. However, if environmental conditions deteriorate, three independent signalling pathways: insulin-like signalling, TGFβ or cGMP signalling can regulate the decision to execute the alternative development pathway and enter dauer state [19, 20]. All three signals are required to block a nuclear hormone receptor from activating the dauer gene expression program. However, loss of any one of the three signals is sufficient to induce *C. elegans* larvae to execute dauer development [21]. These pathways can be manipulated at the molecular or genetic level to specifically induce or suppress dauer formation.

Upon executing dauer development, GSCs undergo a G2/M cell cycle arrest despite the presence of active Notch ligand in the DTCs and GLP-1 expression in the GSCs [15], suggesting that germ cell proliferation is blocked either downstream of, or in parallel to, Notch signalling. The orthologues of LKB1 (*par-4)* and AMPK (the two catalytic subunits *aak-1* and *aak-2*) and PTEN (*daf-18*) co-operate during this stage to trigger the cell cycle quiescence typical of the GSCs during the dauer stage [15]. However, the underlying mechanisms involved in establishing this quiescence are unknown.

To determine what pathways might act downstream of LKB1/AMPK in the establishment and/or maintenance of GSC quiescence, we used dauer-induced germline quiescence as a read out to find genes that modulate these germ cell divisions. Using an unbiased reverse genetic approach, we identified genes that could act as potential targets of LKB1/AMPK signalling that are critical for its role in dauer-associated GSC quiescence and potentially for the tumour suppressor function of LKB1. Since genetic analysis suggests that LKB1/*par-4* must impinge on targets other than AMPK to induce cell cycle arrest, we designed unbiased RNAi-based screens that would favour the identification of genes that act downstream of LKB1/*par-4*, but in a manner that is independent of AMPK. Our data indicate that *par-4* can indeed act independently of AMPK to regulate germline quiescence in the dauer stage. Moreover, most of the genes that act with *par-4,* but do not rely on AMPK to regulate germline quiescence, have documented roles in cell polarity and cytoskeletal regulation. These genes may therefore act downstream of LKB1 such that when LKB1 function is compromised in PJS patients, their misregulation may contribute to the aetiology of the disease.

## Results

**Fig. 1:**
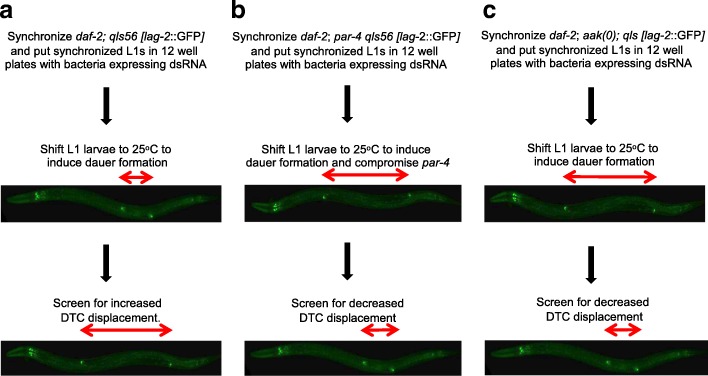
Experimental design for genome-wide RNAi screens to identify genes that interact with LKB1/*par-4* or AMPK/*aak(0)*. All the strains used for the three genome-wide screens contain a temperature-sensitive *daf-2(e1370)* mutation that allowed us to induce dauer by shifting synchronized L1 larvae to 25 °C. Importantly, all the strains carry a *lag-2*::GFP transgene that is expressed in the DTCs, marking the distal extremities of the germ line. This expression allowed us to indirectly evaluate the degree of germline stem cell hyperplasia [15]. **a** The screen was designed to identify genes that result in dauer germline hyperplasia when their function is compromised by RNAi. *daf-2* mutants carrying the *lag-*2::GFP transgene were synchronized and L1 larvae were then put on plates containing IPTG to induce dsRNA expressed from each bacterial clone and were shifted to 25 °C to induce dauer formation. The dauer larvae were subsequently screened for increased DTC displacement; a proxy for germline hyperplasia [15]. The diagrams in **b** and **c** describe screens designed to identify suppressors of *par-4-* and *aak(0)*-induced dauer germline hyperplasia, respectively*.* For **b**, the screen was performed with a strain that contains a temperature-sensitive *par-4* mutation, while in **c** an AMPK null mutant (*aak(0)*) that harboured deletions in both catalytic subunits was used. Upon dauer induction, animals were monitored for reduced displacement between the DTCs indicating suppression of the germline hyperplasia typical of both *par-4* and *aak(0)* mutants. See Methods for more details

To better understand how germline stem cell cycle quiescence is regulated during periods of reduced insulin-like signalling, we performed three independent genome-wide RNAi screens based on feeding dsRNA corresponding to every predicted gene in *C. elegans* [22]. One analysis was designed to isolate genes that result in germline hyperplasia typical of LKB1(*daf-2; par-4)* or AMPK compromise (*daf-2; aak(0)*) during the dauer stage. Two additional genome-wide RNAi surveys were performed to identify genes that, when blocked, would suppress the germline hyperplasia phenotype typical of both LKB1*/par-4* or AMPK mutant dauer larvae. The activity of the identified genes would presumably be under LKB1/PAR-4 and/or AMPK-mediated regulation, and in the absence of either of these genes the activity of these targets would go unchecked during the dauer stage. The results from these two screens could contribute to the identification of novel genes that suppress hyperplasia exclusively in (i) the LKB1/*par-4* mutant background, (ii) the AMPK mutant background, and (iii) in both the *par-4* and AMPK mutant backgrounds. All the strains used for the above mentioned screens express a LAG-2::GFP transgene allowing us to visually assess the distance between the DTCs; a proxy for the degree of germline hyperplasia [15] (Fig. 1).

To eliminate as many false positives as possible from the primary screens, secondary and tertiary screens were performed. Thus, only candidates that demonstrated a reproducible response to the RNAi treatment in each round were considered as the potential candidates. For each RNAi treatment, at least 100 dauer larvae were screened to analyze the distance between the DTCs. As RNAi can exhibit differential phenotypic penetrance, expressivity, and variance, we applied a threshold such that a gene candidate would only qualify for the next round of analysis if greater than 5% of the larvae (as 1–5% animals treated with an empty RNAi vector displayed DTC displacement) showed detectable DTC displacement. In the event that an RNAi only partially affects the DTC displacement (expressivity), but in more than 5% dauer larvae (penetrance), then it would be retained, as this would still indicate that this gene must somehow affect dauer germline quiescence.

**Fig. 2:**
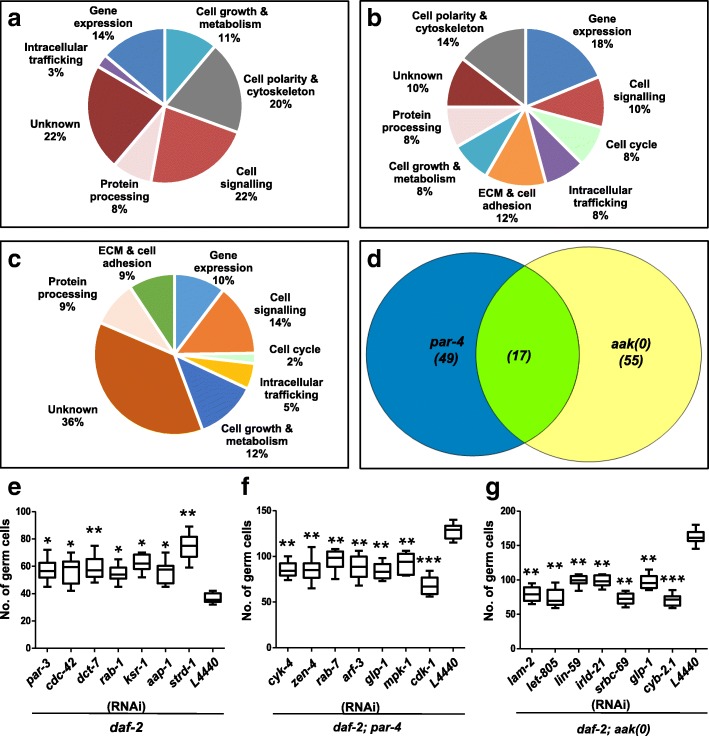
Identification of genes that interact with AMPK/*aak(0)* and LKB1/*par-4* to regulate germline quiescence during the dauer stage. Candidate genes identified in the genome-wide RNAi screens were categorized based on their functional description on WormBase. **a** Gene ontology (GO) terms of genes that phenocopied *par-4* and *aak(0)* mutations and caused germline hyperplasia when subjected to RNAi. **b** and **c** Genes that suppressed *par-4* and *aak(0)* dependent dauer germline hyperplasia respectively were categorized by GO terms and shown in a pie chart. The most common GO terms include genes with known functions in the regulation of gene expression, DNA and RNA metabolism; in cytoskeletal regulation and cell polarity; in the regulation of cell-ECM adhesion and ECM integrity; cell signal transduction; cell cycle progression; cell growth signalling and metabolism; intracellular trafficking and protein processing. A significant portion of candidate genes remain uncharacterized and fall into the Unknown functional group. **d** A Venn diagram that illustrates the total number of RNAi candidate genes that suppress the hyperplasia in the *par-4* and *aak(0)* backgrounds. The overlap (green) represents the number of candidates that suppressed the hyperplasia in both genotypes, implying that they act downstream of PAR-4 in an AMPK-dependent manner. **e** To validate the candidates identified that phenocopy the hyperplasia observed in *par-4* and AMPK mutants, *daf-2(e1370)* dauer larvae were subjected to RNAi against genes representing different functional classes. The germ cell nuclei counts were performed following DAPI staining. RNAi against the LKB1 pseudokinase component *strd-1* was used as a positive control. **f**, **g** To validate the identified gene candidates and confirm the suppression of *par-4* and *aak(0)* dependent dauer germline hyperplasia, genes from different functional categories were depleted using RNAi and germ cell nuclei counts were performed on extruded DAPI-stained gonads. RNAi of these genes resulted in a significant decrease in the germline hyperplasia typical of *par-4* and *aak(0*) mutant dauer larvae. ***P < 0.0001, **P < 0.001, *P < 0.05 when compared to L4440 using two-tailed *t-test*, *n* = 50

From our genome-wide analysis, we identified a total of 39 genes that resulted in dauer germline hyperplasia when they were subjected to feeding RNAi (Table 1). The suppressor screens that were carried out for *par-4* and *aak(0)* allowed us to identify 49 and 55 candidate genes, respectively; all of which resulted in a pronounced and reproducible reduction in dauer germline hyperplasia when mutant animals were fed the corresponding dsRNA (Tables 2 and 3). The candidate genes were classified according to their predicted roles in diverse biological processes using Gene Ontology (GO) annotation [23, 24]. The most prevalent of these-included regulation of cell polarity and/or the cytoskeleton, nutrient signalling and metabolism, tumour suppressor genes, intracellular trafficking regulation, the extracellular matrix (ECM) and cell adhesion, protein processing, gene expression regulation and lastly, a significant number of genes with unknown function (Fig. 2a-c).

**Table 1:** Candidate genes that resulted in germline hyperplasia during the dauer stage following RNAi

Chromosome	Cosmid identifier	Gene	Brief description
I	ZC123.3		Homeobox protein; homologue to human ATBF1 transcription factor and tumour suppressor
I	Y110A7A.10^a^	*aap-1*	PI3K p50/55 adaptor/regulatory subunit orthologue
I	T08B2.10	*rps-17*	40S ribosomal protein S17
I	F57B10.8		Activator of basal transcription
I	F26E4.4^a^		Activator of basal transcription
I	T06G6.9^a^	*pfd-3*	Molecular chaperon Prefoldin, subunit 3; orthologue to human VHL-binding protein tumour suppressor
I	Y87G2A.h^a^	*gpi-1*	Putative glucose 6-phosphate isomerase; Required for normally short lifespan
II	C23H3.4^a^	*sptl-1*	Putative serine palmitoyltransferase
II	F53A10.2		Rap-1 GTPase-activating protein; orthologue to a human tumour suppressor in squamous cell carcinoma
II	T05C1.6^a^		Calmodulin-binding transcriptional activator
II	R07G3.1	*cdc-42*	RHO small GTPase; regulator of polarity
II	T05C12.6	*mig-5*	One of three homologues to Dishevelled in *Drosophila*
II	T22C8.2	*chhy-1*	Chondroitin hydrolase; similar to human HYAL1, a potential tumour suppressor in lung cancer cell lines
II	F40F8.9^a^	*lsm-1*	Small nuclear ribonucleoprotein splicing factor
II	T06D8.2^a^		No description available
II	C50E10.3^a^	*sre-53*	G protein-coupled chemoreceptor
II	W01D2.2	*nhr-61*	Nuclear hormone receptor
III	R74.2^a^		No description available
III	Y44F5A.1		Protein containing WD40 repeats; required for protein-protein interactions
III	C27F2.4^a^		Predicted protein carboxyl methylase
III	R02F2.7^a^		No description available
III	F54E7.3	*par-3*	PDZ-domain containing protein; required for polarization of the early embryo
III	B0336.3^a^		Protein containing and RNA recognition motif
III	C07H6.5	*cgh-1*	Putative RNA helicase; inhibits apoptosis in oocytes
III	C50C3.9^a^	*unc-36*	Voltage-dependent calcium ion channe
III	Y52D3.1	*strd-1*	Activator of LKB1
IV	F15E6.8	*dct-7*	RNA binding protein controlled by DAF-16/FOXO; affects germline tumours
IV	H35B03.2^a^		Subunit of nuclear ribonuclease P
IV	C06E7.1^a^	*sams-3*	S-adenosylmethionine synthetase
IV	F12F6.5	*sgrp-1*	Homologue to Cdc42-interacting protein CIP4
IV	Y51H4A.c	*rho-1*	Rho GTPase; required for actin filament-based processes including embryonic polarity
V	F48G7.9		Serine/threonine protein kinase
V	C39F7.4	*rab-1*	Ras-like GTPase, orthologue to Rab1
V	T03D3.11^a^	*srj-44*	7-transmembrane olfactory receptor
V	F26D11.11^a^	*let-413*	Localizes to basolateral region of epithelial cells and required for adherens junction formation; strong homology to *Drosophila* Scribble
X	C14A11.3	*cgef-1*	Guanosine nucleotide exchange factor for Rho and Rac GTPases
X	C38C5.1^a^		No description available
X	F13B9.5	*ksr-1*	Kinase suppressor of Ras
X	T09B9.3^a^		Glycerophosphoryl diester phosphodiesterase

The gene identity of each RNAi clone was determined by the database available with the commercially available Ahringer RNAi library. The brief descriptions for each gene locus are derived from WormBase or determined according to their functional homology

^a^RNA candidates that potentially function germline non-autonomously

**Table 2:** Candidate genes that suppress *par*-*4*-dependent hyperplasia during the dauer stage

Chromo-some	Cosmid identifier	Gene	Brief description
I	H26D21.1^a^	*hus-1*	DNA damage checkpoint protein required for DNA damage-induced cell cycle arrest in *C. elegans*
I	C43E11.6	*nab-1*	Neurabin orthologue that regulate interactions between actin and microtubules during cell division, migration and growth cone guidance
I	F27C1.7^a^	*atp-3*	Subunit of mitochondrial ATP synthase, which regulates growth rate, body size, and ageing.
I	C34G6.6^a^	*noah-1*	ECM component required for molting, development, vulval development, and normal body morphology
I	H15N14.1	*adr-1*	Adenosine deaminase that acts on RNA by deaminating adenosines and generating inosines in dsRNA; protects transgenic RNA from RNAi silencing
I	C54G4.8^a^	*cyc-1*	Subunit of complex III cytochrome c reductase required for normal ATP production
I	F26E4.9^a^	*cco-1*	Subunit of cytochrome c oxidase-1, a component the electron transport chain in mitochondria
I	E03H4.8^a^		Uncharacterized
I	H28O16.1^a^		Uncharacterized
I	M01D7.1^a^		Uncharacterized
I	ZK1151.1	*vab-10*	A spectraplakin, component of the hemidesmosome in *C. elegans;* required for transducing mechanical signals from muscle cells to epidermis, and DTC migration
I	B0414.2	*rnt-1*	Transcription factor required for seam cell proliferation; interacts with SMA-4 and regulates expression of CDK- inhibitor *cki-1*
II	T15H9.3^a^	*hlh-6*	Helix loop helix transcription factor
II	C47D12.6^a^	*tars-1*	Threonyl amino-acyl tRNA synthetase
II	ZK930.3^a^	*vab-23*	Transcription factor involved in ventral closure, elongation
II	W08F4.6^a^	*mlt-8*	Putative signalling peptide secreted from cells involved in the L2/L3 molting process
II	W03C9.3	*rab-7*	Rab-GTPase required for endosome to lysosome trafficking
II	F59G1.3^a^	*vps-35*	Vacuolar protein sorting factor
II	R09D1.7^a^	*chil-20*	Chitinase-like protein
II	C41C4.4^a^	*ire-1*	Transmembrane serine/threonine kinase and endoribonuclease necessary for unfolded protein response (UPR)
II	F10B5.1^a^	*rpl-10*	Ribosomal subunit L10
III	T05G5.3	*cdk-1*	Cyclin-dependent kinase required for cell cycle progression through the G2/M checkpoint
III	R01H10.1^a^	*div-1*	DNA polymerase subunit required for normal interphase timing and asymmetric distribution of PIE-1 and P granules
III	K12H4.4		Uncharacterized
III	C04D8.1	*pac-1*	Rho-GAP involved in establishing radial asymmetry during *C. elegans* development by regulating the spatial localization of CDC-42
III	F43C1.2^a^	*mpk-1*	Mitogen-activated-protein-kinase
III	F58A4.8	*tbg-1*	γ-tubulin
III	K08E3.6	*cyk-4*	Rho-GAP and member of the centralspindlin complex required for cytokinesis; also factor localized to intercellular bridge in the rachis required for gonad structural integrity
III	R10E11.2^a^	*vha-2*	V-ATPase subunit involved in protein sorting and receptor mediated endocytosis
III	F26A1.14^a^		Uncharacterized
III	H19M22.2^a^	*let-805*	Myotactin; subunit of the hemidesmosome complex
III	F26F4.10	*rars-1*	Arginyl-tRNA synthetase
III	F02A9.6	*glp-1*	Notch receptor homologue and required for germline proliferation
III	M88.6	*pan-1*	Transmembrane protein required for completion of larval molts; expression enriched in the germ line
III	B0336.2	*arf-1.2*	ADP-ribosylation factor homolog, a GTPase that regulates intracellular trafficking and the actin cytoskeleton
III	T12A2.2^a^	*stt-3*	Yeast oligosaccharyltransferase subunit homologue
III	F37C12.4	*rpl-36*	Large ribosomal subunit L36
III	F58A4.11^a^	*gei-13*	Predicted DNA binding protein involved in body shape regulation, cuticle synthesis and locomotion
IV	C46A5.2	*del-7*	Degenerin-like protein; degenerin family of proteins are sodium ion channels essential for homeostasis and involved in mechanotransduction
IV	F56H11.1^a^	*fbl-1*	Fibulin, a component of the extracellular matrix required for DTC migration initiation
IV	F56H9.5^a^	*lin-25*	Subunit of the Mediator complex, which functions downstream of LET-60 to regulate differentiation of a number of cell types
IV	F57H12.1	*arf-3*	ADP-ribosylation factor homolog, a GTPase that regulates intracellular trafficking and the actin cytoskeleton
IV	R13H7.1^a^	*srx-20*	Serpentine receptor, class X
V	K06A4.3	*gsnl-1*	Gelsolin-related protein predicted to function as an actin regulatory protein, capping barbed ends of actin filaments
V	Y49A3A.2^a^		Uncharacterized
V	F33E11.1^a^	*nhr-15*	A nuclear hormone receptor
V	E02C12.3^a^	*srx-47*	Serpentine receptor, class X
V	F53A9.10^a^	*tnt-2*	Troponin, a tropomyosin binding protein
V	C54D1.5^a^	*lam-2*	Subunit of laminin required for basement membrane integrity and gonad morphology

The gene identity of each RNAi clone was determined by the database available with the commercially available Ahringer RNAi library. The brief descriptions for each gene locus are derived from WormBase or determined according to their functional homology

^a^RNA candidates that potentially function germline non-autonomously

**Table 3:** Candidate genes that suppress AMPK-dependent hyperplasia during the dauer stage

Chromo-some	Cosmid identifier	Gene	Brief description
I	T12F5.4	lin-59	SET domain-containing protein, closely related to *Drosophila* tri-thorax ASH1 protein implicated in chromatin remodeling.
I	H15N14.1^a^	adr-1	Adenosine deaminase that acts on RNA by deaminating adenosines and generating inosines in dsRNA; protects transgenic RNA from RNAi silencing
I	C54G4.8^a^	cyc-1	Subunit of complex III cytochrome c reductase required for normal ATP production.
I	F26E4.9^a^	cco-1	Subunit of cytochrome c oxidase-1, a component of the electron transport chain in mitochondria
I	F35C12.1^a^		Uncharacterized
I	I-5 Q4 G9^b^		A *vab-10* isoform. A spectraplakin, component of the hemidesmosome in *C. elegans;* required for transducing mechanical signals from the muscle cells to epidermis
I	E03H4.8		Uncharacterized
I	H28O16.1^a^		Uncharacterized
I	Y18D10A.13	pad-1	Unfamiliar conserved protein required for embryonic development
II	T08E11.5^a^	fbxc-19	F-box C protein
II	B0281.6		Uncharacterized
II	F42G2.4^a^	fbxa-182	F-box C protein
II	K02F6.1		Uncharacterized
II	T24E12.9^a^		Uncharacterized
II	F29G1.3^a^	vps-35	Vacuolar protein sorting factor
II	T02G5.9^a^	kars-1	Lysyl(K) Amino-acyl tRNA Synthetase
II	F22B5.2	eif-3.G	Encodes a homologue of eukaryotic translation initiation factor 3, subunit 4. Affects embryonic viability, fertility and growth
II	C50E10.2^a^		Uncharacterized
II	C50E10.3^a^	sre-53	Serpentine receptor, Class E
II	Y53F4B.g		Uncharacterized
III	H19M22.2^a^	let-805	Myoactin; subunit of the hemidesmosome complex
III	T20B6.3		Uncharacterized
III	F26A1.13^a^		Uncharacterized
III	B0336.2	arf-1.2	ADP-ribosylation factor homologue, a GTPase that regulates intracellular trafficking and the actin cytoskeleton
III	C28H8.11^a^	tdo-2	Tryptophan 2,3-DiOxygenase
III	T12A2.2^a^	stt-3	Yeast oligosaccharyltransferase subunit homologue
III	C18F10.4^a^	srg-1	Serpentine Receptor, Class G
III	F23F12.6	rpt-3	A triple A ATPase subunit of the 26S proetosome’s 19S regulatory particle base subcomplex; functions as a reverse chaperone by unfolding substrates and translocating them into the core proteolytic particle (CP) of proteasome
III	R13A5.7^a^		Uncharacterized
III	K12H4.4^a^		Uncharacterized
III	F02A9.6	glp-1	Notch receptor homologue required for germline stem cell mitotic proliferation
III	F54G8.1^a^	irld-34	Insulin/EGF-receptor L Domain protein
III	R10E11.2^a^	vha-2	V-ATPase subunit involved in protein sorting and receptor-mediated endocytosis
IV	F47C12.6		Uncharacterized
IV	W03B1.6^a^	oac-51	O-ACyltransferase homologue
IV	F57H12.1	arf-3	ADP-ribosylation factor homologue, a GTPase that regulates intracellular trafficking and the actin cytoskeleton
IV	Y43E12A.1	cyb-2.1	A cyclin B isoform
IV	C28C12.11^a^		Uncharacterized
IV	K07F5.7		Uncharacterized
IV	C04G2.11^a^	irld-21	Insulin/EGF-receptor L Domain protein
IV	T17B5.1^a^		Uncharacterized
IV	F25H8.3	gon-1	A metalloprotease involved in ECM degradation; controls gonadal morphogenesis
V	F33E11.1^a^	nhr-15	A nuclear hormone receptor
V	F48G7.11^a^	nhr-190	A nuclear hormone receptor
V	C24B9.7^a^	srg-59	Serpentine Receptor, Class G
V	F35F10.3		Uncharacterized
V	F32D1.2	hpo-18	Hypersensitive to POre-forming toxin
V	T24A6.12^a^	srbc-69	Serpentine Receptor, Class BC
V	Y47D7A_1^a^43.d		Uncharacterized
V	C10G8.8^a^		Uncharacterized
V	F07G11.4		Uncharacterized
V	C54F6.10^a^	str-31	Seven-transmembrane G-protein coupled receptor
V	C12D5.7^a^	cyp-33A1	Cytochrome P450 family
V	C37C6.6^a^	mig-6	Similar to extracellular matrix proteins papilin and lacunin; required for DTC migration at all phases
X	C54D1.5^a^	lam-2	Subunit of laminin required for basement membrane integrity and gonad morphology

The gene identity of each RNAi clone was determined by the database available with the commercially available Ahringer RNAi library. The brief descriptions for each gene locus are derived from WormBase or determined according to their functional homology

^a^RNA candidates that potentially function germline non-autonomously

^b^These wells did not appear to be in the Ahringer library database. The RNAi clone from this well was sequenced and compared to other species to identify potential orthologues

To further validate our candidates, dauer germ cell quantification was performed upon RNAi of each individual candidate gene identified in all the screens to confirm their role in maintaining dauer germline quiescence (Fig. 2e-g, subset of genes shown). To verify that the identified candidate genes from the screens were not specific to the disruption of insulin-like signalling, but rather, their activity is required in other signalling pathways that control dauer formation, we quantified the number of germ cell nuclei and validated the function of each candidate gene identified in mutants with compromised TGF-β signalling (*daf-7)* (Tables 1, 2 and 3). We observed no differences in RNAi-mediated phenotypes suggesting that each of the identified genes was required for germline quiescence downstream of at least two different triggers for dauer formation.

Genetic evidence suggests that *par-4* acts to suppress the germline proliferation in a manner that is, at least in part, independent of AMPK [15]. We therefore compared the candidate genes that suppressed *par-4*-dependent dauer germline hyperplasia and those that suppressed the hyperplasia in the *aak(0)* background. If the two genes function in a linear pathway where PAR-4 activates AMPK to phosphorylate critical targets involved in cell cycle quiescence, the genes that were identified in the two independent suppressor screens should be identical. This was however not what we observed. Our analysis revealed that only 17 candidates were common to both data sets, implying that PAR-4 mediates its control over cell proliferation via AMPK through these targets (Fig. 2d). More importantly, the remaining candidates must act downstream of, or in parallel to, PAR-4. These genes may also act independently of AMPK, since they do not affect the hyperplasia caused by the loss of AMPK signalling.

The GO term analysis suggests that the majority of the genes that act exclusively downstream of LKB1/*par-4* have documented roles in cytoskeletal regulation and cell polarity (Table 4). This is somewhat counterintuitive given that the cytoskeletal profile of mitotic germ cells in *C. elegans* remains elusive, while germ cell polarity, particularly with respect to the apical/basal axis, must be quite unique given the syncytial nature of germ cells within the gonad.

**Table 4:** Candidate genes that exclusively suppress *par-4-*dependent hyperplasia during the dauer stage

Chromo-some	Cosmid identifier	Gene	Brief description
I	H26D21.1	*hus-1*	DNA damage checkpoint protein required for DNA damage-induced cell cycle arrest in *C. elegans*
I	C43E11.6	*nab-1*	Neurabin orthologue that regulate interactions between actin and microtubules during cell division, migration and growth cone guidance
I	F27C1.7	*atp-3*	Subunit of mitochondrial ATP synthase, which regulates growth rate, body size, and ageing
I	C34G6.6	*noah-1*	ECM component required for molting, development, vulval development, and normal body morphology
I	M01D7.1		Uncharacterized
I	B0414.2	*rnt-1*	Transcription factor required for seam cell proliferation; interacts with SMA-4 and regulates expression of CDK- inhibitor *cki-1*
I	T05F1.6	*hsr-9*	Cell cycle checkpoint protein in response to DNA damage
II	T15H9.3	*hlh-6*	Helix loop helix transcription factor
II	R09D1.7	*chil-20*	Chitinase-like protein
II	C47D12.6	*tars-1*	Threonyl amino-acyl tRNA synthetase
II	ZK930.3	*vab-23*	Transcription factor involved in ventral closure, elongation
II	W03C9.3	*rab-7*	Rab-GTPase required for endosome to lysosome trafficking
II	F10B5.1	*rpl-10*	Ribosomal subunit L10
II	W08F4.6	*mlt-8*	Putative signalling peptide secreted from cells involved in the L2/L3 molting process
II	C41C4.4	*ire-1*	Transmembrane serine/threonine kinase and endoribonuclease necessary for unfolded protein response (UPR).
III	T05G5.3	*cdk-1*	Cyclin-dependent kinase required for cell cycle progression through the G2/M checkpoint
III	R01H10.1	*div-1*	DNA polymerase subunit required for normal interphase timing and asymmetric distribution of PIE-1 and P granules
III	K12H4.4		Uncharacterized
III	C04D8.1	*pac-1*	Rho-GAP involved in establishing radial asymmetry during *C. elegans* development by regulating the spatial localization of CDC-42
III	F43C1.2	*mpk-1*	Mitogen-activated-protein-kinase
III	F58A4.8	*tbg-1*	γ-tubulin
III	K08E3.6	*cyk-4*	Rho-GAP and member of the centralspindlin complex required for cytokinesis; also factor localized to intercellular bridge in the rachis required for gonad structural integrity
III	F26F4.10	*rars-1*	Arginyl-tRNA synthetase
III	M88.6	*pan-1*	Transmembrane protein required for completion of larval molts; expression enriched in the germ line
III	T12A2.2	*stt-3*	Yeast oligosaccharyltransferase subunit homologue
III	F37C12.4	*rpl-36*	Large ribosomal subunit L36
III	F58A4.11	*gei-13*	Predicted DNA binding protein involved in body shape regulation, cuticle synthesis, and locomotion
IV	C46A5.2	*del-7*	Degenerin-like protein; degenerin family of proteins are sodium ion channels essential for homeostasis and involved in mechanotransduction
IV	F56H11.1	*fbl-1*	Fibulin, a component of the extracellular matrix required for DTC migration initiation
IV	F56H9.5	*lin-25*	Subunit of the Mediator complex, which functions downstream of LET-60 to regulate differentiation of a number of cell types
IV	R13H7.1	*srx-20*	Serpentine receptor, class X
V	K06A4.3	*gsnl-1*	Gelsolin-related protein predicted to function as an actin regulatory protein, capping barbed ends of actin filaments

The gene identity of each RNAi clone was determined by the database available with the commercially available Ahringer RNAi library. The brief descriptions for each gene locus are derived from WormBase or determined according to their functional homology

### Genes involved in cytoskeleton and polarity

The tumour-like effects of disrupting apico-basal cell polarity has been well documented in *Drosophila melanogaster* where mutations that affect cell polarity result in severe hyperplasia of the affected somatic tissues [25]. 18% of the genes that caused the germline hyperplasia and 14% of the candidates from our *par-4* suppressor screen belonged to the subset of genes that affect these processes, respectively. This number is surprisingly high considering that no apical/basal polarity has been previously ascribed to the *C. elegans* germ cells.

Interestingly, compromising the function of the Partitioning defective gene *par-3* resulted in the germline hyperplasia typical of *par-4* and *aak(0)* mutants. *Par-3* has a well characterized role in the polarization of the early *C. elegans* embryo where it is required for the initial establishment of anterior-posterior polarity and the segregation of the germline determinants in the one cell zygote [26–28]. In mammalian cells PAR-3 acts at the apical region of epithelial cells to distinguish this region from the basolateral cortex [29].

In *C. elegans* PAR-3 forms a complex at the anterior cortex of the zygote with PAR-6 and PKC-3, which excludes PAR-1 and PAR-2 to the posterior cortex [30]. Formation of the anterior and posterior PAR complexes is essential to specify the germline blastomere [31–33]. Later in development, the PAR-3/PAR-6/aPKC-3 complex localizes to the apical cortex of epithelial cells and is required for the maintenance of adherens junctions (known as apical junctions in *C. elegans*) [34, 35].

**Fig. 3:**
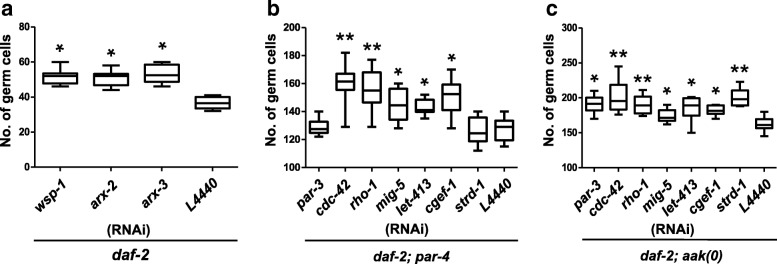
Compromise of both cell polarity and actin cytoskeletal regulators results in dauer germline hyperplasia. **a** Canonical actin cytoskeleton regulators which were not identified in the screen were depleted using RNAi and germ cell nuclei counts were performed. Depletion of *wsp-1, arx-2, arx-3* resulted in significant increase in the number of germ cells. **b** and **c** To test if *par-4* mediates cell polarity independently of AMPK to establish dauer GSC quiescence, genes belonging to the cell polarity and cytoskeleton category were depleted in either a *par-4* or an *aak(0)* background and germ cell nuclei counts were performed. **P < 0.001, *P < 0.05 when compared to L4440 using two-tailed *t- test*, *n* = 50

To determine if *par-3* acts with *par-4* in an AMPK-dependent or -independent manner, we compromised *par-3* in both *par-4* and the *aak(0)* mutant animals. Loss of *par-3* was not additive to the hyperplasia caused by loss of *par-4* based on the germ cell numbers that were present in the compound mutants. On the other hand, loss of *par-3* was indeed additive in the *aak(0)* background (Fig. 3b, c). This suggests that *par-3* and *par-4* function together in a linear genetic pathway, yet it acts independently of *aak(0)*/AMPK to affect germ cell quiescence in the *C. elegans* dauer larva.

Another set of genes required for GSC quiescence include *mig*-*5* and *let*-*413*, which are homologues of the *Drosophila* tumour suppressor genes Dishevelled and Scribble, respectively. Dishevelled is a key regulator of planar cell polarity (PCP) in imaginal disc epithelia [36–38], while Scribble is part of a protein complex, along with Lethal giant larvae (Lgl) and Discs-large (Dlg), that defines the basolateral domain of epithelial cells [39, 40]. Our genetic analyses suggest that these genes, which we identified in our genetic screen, act independently of *par-4* or *aak(0),* since they both enhance germline hyperplasia when compromised in either the *par-4* or the *aak(0)* background (Fig. 3b, c).

Two other candidates in this category include *cdc*-*42* and *rho-1*, small Rho-like GTPases with many well characterized functions, including polarization of the early embryo, tight junction assembly, acto-myosin contractility, and endocytosis [41–44]. Specifically, together with *rho-1*, *cdc-42* is required for the initial actinomyosin contractions of the *C. elegans* one-cell embryo to generate a cortical flow of proteins, which is the first asymmetry-generating event in development [44]. CDC-42-dependent activation of WASP (Wiskott-Aldrich Syndrome Protein) is essential to activate the Arp2/3 complex and consequently stimulate actin assembly [45].

To determine if *cdc-42* impinges on this pathway and whether actin cytoskeletal assembly is vital to establish GSC quiescence in dauer larvae, we performed RNAi experiments against *wsp-1* (*C. elegans* WASP orthologue) and *arx-2* and *arx-3;* the *C. elegans* orthologues of the Arp2/3 complex subunits followed by quantification of the resulting germ cell numbers. Disruption of either the WASP or the Arp2/3 complex resulted in significant increase in the germ cell counts (Fig. 3a), suggesting that *cdc-42* activity is essential for the maintenance of germline stem cell quiescence during the dauer stage, presumably through its role in regulating actin cytoskeletal organization. Furthermore, when *cdc-42* or *rho-1* are compromised in either a *par-4* or an *aak(0)* mutant, we observed an additive increase in germ cell counts (Fig. 3b, c), typical of these two gene products acting, most likely together, in an independent pathway that regulates germline quiescence in concert with AMPK and LKB1 during the dauer stage.

Among the collection of genes that suppress the *par-4*-dependent hyperplasia in dauer larvae, 7 identified genes are involved in regulating the cytoskeleton and cell polarity. Of particular interest are *cyk-4* and *pac-1*; two Rho-GAPs that regulate cytoskeletal dynamics by deactivating Rho-GTPases [46]. Since at least two small GTPases were identified in our genetic screen for genes that resulted in dauer germline hyperplasia, the discovery that their inactivation rescues hyperplasia in a *par-4* background strongly suggests that a mechanism involving the Rho-family GTPases is required to establish or maintain quiescence in the germ line during the dauer stage.

The RhoGAP-encoding *cyk-4* is a conserved component of Centralspindlin, a complex that lies at the heart of central spindle assembly and cytokinesis in metazoans [47–49]. Recently, a novel role of *cyk-4* that is independent of its function in cytokinesis was demonstrated through its ability to regulate germline architecture by maintaining intracellular bridges between the germ cells in *C. elegans* [50]. Intercellular bridges (or rachis bridges) are formed and stabilized along the distal gonad arm between the individual germ cells and the rachis. Rachis bridges therefore resemble the mitotic cleavage furrow and actinomyosin ring, retaining many of the factors found in these structures [50, 51].

To determine if other components of the Centralspindlin complex cooperate with CYK-4 to regulate germline quiescence during the dauer stage we used RNAi to disable a second Centralspindlin component, *zen-4,* which belongs to the kinesin-6 subfamily of plus end directed microtubule motor proteins [52]. *zen-4*(RNAi) significantly suppressed *par-4-*dependent hyperplasia (Fig. 2f), suggesting that *par-4* may impinge upon the Centralspindlin complex to either affect its assembly, stability, and/or function, either directly or indirectly, to mediate dauer GSC quiescence. The pseudokinase adaptor protein STRAD is differentially required to enhance the activity of LKB1/PAR-4 and is essential to establish dauer germline quiescence [53]. To test if *strd-1* is required for the AMPK-independent function of *par-4* to establish dauer germline quiescence, we compromised its function in both *par-4* and *aak(0)* mutants and quantified the number of germ cells in the resulting dauer gonads. Based on our results, *strd-1* acts in a *par-4-*dependent pathway that functions in parallel to AMPK (Fig. 3b, c).

Interestingly, the candidate genes that we isolated through the *par-4* suppressor screen that are involved in cell polarity and cytoskeletal regulation did not suppress the germline hyperplasia typical of *aak(0)* mutant dauer larvae (Table 4). This is consistent with our genetic analysis that indicated that the germ cell numbers observed in *par-4; aak(0)* mutant dauer are significantly greater than in *aak(0)* mutant animals alone [15]. Therefore, PAR-4 likely regulates germline quiescence through its ability to control cellular mechanisms that are both dependent and independent of AMPK kinase targets during the dauer stage in *C. elegans*. These AMPK-independent targets suggest that PAR-4 must interact with key regulators of cell polarity and cytoskeletal dynamics.

### *par-4* mutants show temporal defects in actin organization at the rachis-adjacent membrane independently of AMPK

Because we identified a surprisingly high number of genes that act in cytoskeletal and cell polarity organization, we wondered whether the loss of *par-4* function might affect the actin cytoskeletal network and consequently permit the germ cells to undergo supernumerary cell divisions during the dauer stage. To examine the changes in cytoskeletal profiles, we monitored actin cytoskeletal organization in the dauer quiescent germ line and how it is impacted by the absence of PAR-4 and/or AMPK.

**Fig. 4:**
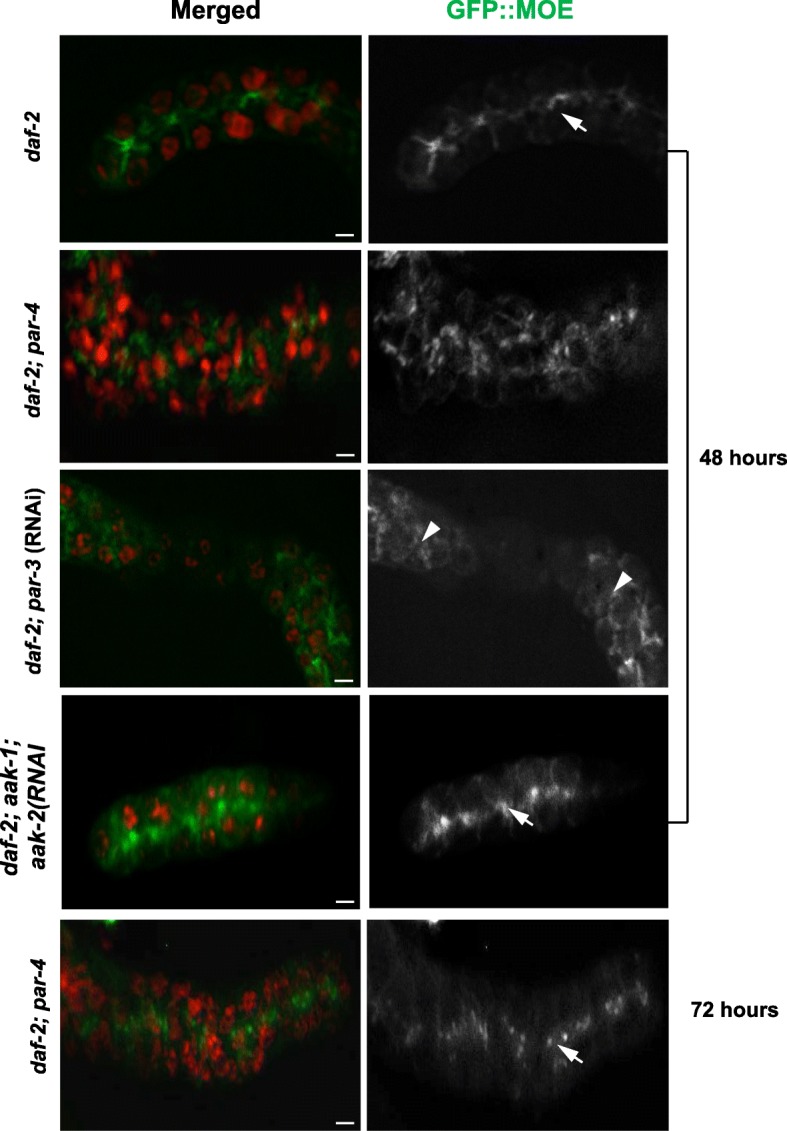
The organization of the actin cytoskeleton is initially perturbed but is resolved later in the *par-4* mutant dauer germline. D*af-2(e1370)* dauer larvae that express a GFP reporter that marks the actin cytoskeleton were examined to monitor any change to the actin cytoskeletal network in the germ line. All images in the left panel are merged, condensed Z stacks. Actin filaments are organized at the membrane adjacent to the rachis in the *daf-2* dauer germ line (arrow) at 48 h after shifting to the restrictive temperature of 25 °C. Actin organization is perturbed (arrowhead) in the germ line of *par-4* dauer larvae at 48 h, but is resolved at 72 h (arrow). Similarly, *par-3* compromised dauer larvae exhibit defects in actin organization at the rachis-adjacent membrane. No defects were observed in the actin organization at the rachis-adjacent membrane in the *daf-2; aak-1; aak-2*(RNAi) treated dauer germ line at 48 h (arrow). *n* = 15, scale bar: 4 μm

Using a *daf-2(e1370)* strain that harbours a transgenic reporter *Ppie-1::*GFP::MOE; a fusion protein that decorates actin filaments with GFP and recapitulates the distribution of F-actin in vivo specifically in the germ line [54], we studied the changes in the actin cytoskeletal re-arrangements within the larval germ line at varying time intervals following the initiation of dauer development. In the quiescent germ line of most dauer larvae the actin filaments are localized at the membrane adjacent to the rachis, but in the *par-4* mutant dauer germline actin organization at the membrane adjacent to the rachis is temporally perturbed. At 48 h after switching to their restrictive temperature of 25 °C, actin filaments are completely disorganized, but at 72 h the disorganization of the network is resolved and appears organized, similar to *daf-2* dauer larvae (Fig. 4). These observations suggest that the early re-organization of the actin cytoskeleton that occurs in the dauer germ line is controlled by *par-4*, but later during the dauer stage other regulators that control the cytoskeletal arrangement become active and can compensate in its absence to re-organize the actin network.

No obvious temporal or spatial defects in cytoskeletal arrangement were observed in *daf-2; aak-1* dauer larvae treated with *aak-2* (RNAi). Actin filaments localized at the membrane adjacent to the rachis in a configuration very similar to *daf-2* mutants (Fig. 4). Therefore, *par-4* regulates actin organization at the rachis bridge during the early phase of the dauer diapause, which may contribute to the establishment of quiescence in the dauer germ line. Most importantly however, it does this independently of AMPK.

Based on our genetic analysis, we propose that *par-4* may require *par-3* in an AMPK-independent manner to establish quiescence in the dauer germ line. To confirm if PAR-3 is essential to regulate temporal actin cytoskeletal organization, we treated *daf-2* animals with *par-3*(RNAi) and monitored actin cytoskeletal organization in the dauer germ line. Similar to *par-4* mutants, compromise of *par-3* results in temporal defects in the arrangement of actin at the rachis-adjacent membrane within the dauer germ line (Fig. 4). When examined in light of previous data obtained in the one cell zygote, these data suggest that PAR-3 may be one of the downstream targets of PAR-4 required to mediate temporal cytoskeletal changes to ensure that germ cells undergo quiescence in response to dauer cues. However, our data cannot rule out that PAR-3 could function upstream of PAR-4.

Compromise of *cdc-42* also resulted in perturbed actin cytoskeletal arrangement within the dauer germ line (Additional file 1: Figure S1) thus confirming that appropriate cytoskeletal re-organization is crucial to maintain the quiescent state of the germ line typical of the dauer larva and mis-regulation of such cytoskeletal rearrangements at the onset of dauer formation results in aberrant germ cell proliferation.

### Germline autonomous vs non-autonomous functions

The germ line is highly responsive to environmental conditions and the germ cells will alter their cell divisions to meet contingencies that reflect the growth status of the organism [55]. The signals that impinge on the germ cells to regulate these decisions can be transduced either autonomously by sensing energy restriction, or alternatively, signals from the soma could be eventually transmitted to the germ line in a non-autonomous manner to control processes involved in their proliferation or apoptosis [56, 57].

To determine whether the candidate genes we identified in our screens exert their function in a germline autonomous fashion, we tested them in an *rrf-1 (pk1417)* background. *Rrf-1* encodes one of the RNA-dependent RNA polymerases in *C. elegans* that acts in a tissue-specific manner where it is required for the amplification of dsRNA in somatic tissues. Mutations in *rrf-1* result in a significant reduction in somatic RNAi, leaving germline-specific RNAi intact [58, 59]. Although some somatic tissues do remain somewhat RNAi proficient, despite these limitations, this method provides a reasonable approach to test large gene sets to understand whether gene function is required in the soma or the germ line.

By performing feeding RNAi directed to all our candidate genes that resulted in dauer germline hyperplasia, we found that 20 of the 39 candidates resulted in germline hyperplasia in *daf-2; rrf-1* dauer larvae (Table 1); 31 out of 49 candidates identified in *par-4* suppressor screen exhibited reduced germline hyperplasia in *rrf-1* compromised *par-4* mutant dauer larvae (Table 2); and 35 out of 55 candidates isolated in *aak(0)* suppressor screen had a germline non-autonomous role to regulate quiescence (Table 3). Cytoskeletal and polarity regulators identified in the genomic screens acted predominantly in a germline autonomous fashion. This suggests that the activity of these genes is important within the germ line to regulate their polarity; a feature that is critical for the establishment or maintenance of quiescence. Other gene categories identified in all the 3 genomic screens which acted in a germline autonomous manner include genes that belong to the categories of intracellular trafficking, cell cycle regulation, ECM regulation, intracellular signalling and gene expression (Tables 1, 2 and 3). Therefore, a considerable number of the candidates we identified in our screens act in a germline non-autonomous manner, suggesting that PAR-4 and AMPK activity might be sufficient in the soma to instruct the germ cells to arrest proliferation and enter a quiescent state in response to energy stress.

## Discussion

In order to withstand long periods of environmental stress, *C. elegans* can execute an alternative developmental stage called “dauer” [60]. The dauer stage is associated with a global cell cycle arrest presumably as a consequence of diverting the available energy resources that might normally fuel the energetically taxing process of cell proliferation to more essential processes required for long term survival [61, 62]. Previous work revealed that AMPK and LKB1/*par-4* mediate this cycle arrest in the dauer germ line [15]. The disruption of either of these genes causes dauer-specific germline hyperplasia, while depletion of both enhances the hyperplasia significantly, suggesting that the genes do not work in a simple linear pathway to establish or maintain germline quiescence [15].

Because of this inconsistency we carried out a series of genome-wide RNAi surveys to obtain a more comprehensive view of the various genes involved in the regulation of germline quiescence during the dauer stage. The analysis would provide a more detailed understanding of how gene products work downstream or in parallel with these two protein kinases to ensure germline cell cycle arrest during this environmentally challenging period. Furthermore, because of the role of these protein kinases in Peutz-Jeghers Syndrome these genes could correspond to potential kinase targets of these enzymes that become misregulated in this disorder.

From our RNAi analysis, we identified a total of 39 genes that resulted in dauer germline hyperplasia and identified 49 and 55 candidate genes that suppressed *par-4* and *aak(0)* dependent germline hyperplasia, respectively. Further analysis of the suppressor screens revealed the subsets of genes that exclusively suppressed the germline hyperplasia in (i) the LKB1/*par-4* mutant background and (ii) the AMPK mutant background. LKB1 dependent activation of AMPK has been confirmed in several different models, but it appears that genes that suppress *aak(0)* dependent hyperplasia fail to suppress the germline hyperplasia in *par-4* mutants. These data are consistent with previous findings indicating that AMPK could be activated by alternative LKB1/PAR-4-independent pathways. Indeed, CaMKK2 is a significant contributor to AMPK activation in specific contexts, particularly in neurons, but also in LKB1-deficient tumour cells [63, 64].

Though RNAi is a powerful genetic tool, it is important to accept the caveats and limitations associated with the method, namely the associated variation in penetrance and expressivity, in addition to the refractory nature of stable protein to the RNAi procedure. Based on this caveat the number of genes we identified is almost certainly an underestimate. Many essential genes that may be involved in maintaining germline quiescence could not be identified due to their role during early stages of embryogenesis [65]. RNAi at the L1 stage may result in the depletion of the newly transcribed mRNA, but it will not affect proteins that have been translated earlier. This may explain why we detected so few genes involved in general cell cycle progression, as many cell cycle regulators are maternally contributed [66]. Based on the systematic RNAi analysis in wild-type animals, 1170 genes demonstrate lethal and sterile RNAi phenotypes [67]. It is likely that many of these could potentially play a role in the maintenance of dauer germline quiescence, but it would require a more laborious screen design to identify them.

While the germ cells of *C. elegans* show no apparent apical/basal polarity, the screen performed to identify genes that could phenocopy that loss of LKB1/AMPK signalling in the germ line, uncovered numerous genes that have well-defined roles in the regulation of apicobasal polarity and/or in regulating the cytoskeleton. This includes the Par gene, *par*-*3*, which has roles in both embryonic and epithelial polarity [28, 30]; *cdc*-*42*, and acts as a master regulator of cell division and polarity, along with regulating diverse cytoskeletal changes [68], as well as the *C. elegans* orthologues of the Scribble and Dishevelled tumour suppressor genes in *Drosophila*, known as *let*-*413* and *mig*-*5* in *C. elegans*, respectively [69, 70]. Moreover, combining the results of the *par-4* and AMPK suppressor screens, we found that candidate genes encoding cytoskeletal and polarity regulators exclusively suppressed the *par-4*-dependent proliferation, with no effect on the hyperplasia observed in the AMPK mutant animals. Given the role of PAR-4 in establishing early embryonic asymmetry and the identification of polarity regulators from the screen for factors required to maintain the germline cell cycle arrest in dauer, we propose a model where PAR-4 establishes or maintains germline quiescence by at least two mechanisms: one that includes its canonical activation/regulation of AMPK, while another that involves the regulation of some aspect of germline cell polarity and cytoskeleton that is independent of AMPK.

It is puzzling that genes with such well-characterized roles in the establishment and maintenance of apical/basal cell or anterior/posterior polarity impinge on *C. elegans* germ cells, which are essentially symmetrical, and show no clear polarization, while also developing in the shared cytoplasm typical of the gonadal syncytium. Interestingly, we show that actin organization at the rachis-adjacent membrane might be the first indication of germ cell-associated polarization. Furthermore, we show that this regulation of the actin cytoskeleton is downstream of PAR-4, but is independent of AMPK.

How might the actin cytoskeletal regulation at the rachis bridge inform germ cell function and affect proliferation? In general, the physical properties of the cytoskeleton can have a profound influence on cell function and therefore contribute to proliferative behaviour [71]. One possibility is that localized actinomyosin contraction near the rachis-adjacent membrane could set up a polarized state, perhaps by altering the cellular milieu by modifying actin-dependent cytoplasmic movements of key determinants that are necessary for the proper establishment of cell quiescence.

Other interesting genes required for the dauer germline quiescence include those that are involved with various aspects of the extracellular matrix (ECM)—either encoding factors that constitute the ECM, or factors that couple the cytoskeletal network to the overlying matrix. Genes identified in this subset were identified as suppressors of both *par-4-* and AMPK-dependent dauer germline hyperplasia. The germ line is enclosed within a basement membrane, with which both the GSCs and DTCs are in direct contact [72]. Dauer-dependent remodeling of the extracellular matrix may convey important cues necessary for stem cell cycle arrest. The ECM may signal to the germline stem cells directly, where the EGF-like repeats on the extracellular domain of GLP-1 could interact with components of the basement membrane [73] affecting its activity. Alternatively, changes in ECM composition may affect the migration of DTCs, which could in turn affect germline stem cell proliferation. In *C. elegans* the hemidesmosomes anchor cells to the matrix and have recently been shown to be involved in mechanotransduction [74, 75]. The identification as *vab-10* as a germline autonomous gene may suggest that a process involving mechanosensing between the DTCs and the germ cells is important for germ cell cycle regulation; germline stem cell proliferation may be coordinated with DTC migration, or lack thereof.

A number of genes found to be required for germline stem cell quiescence during dauer have demonstrated roles in nutrient signalling and metabolism. These genes were of interest because of the implications of the TOR-mediated growth signalling pathway in the maintenance of germline quiescence during dauer. The genes which resulted in germline hyperplasia upon their RNAi-mediated knockdown are *aap-1* (AGE-1 adaptor protein), *gpi-1* (glucose phosphate isomerase), *sptl-1*(serine palmitoyltransferase) and *sams-3* (S-adenosylmethionine synthetase). Mutations in either of *aap-1* or *gpi-1* result in extended lifespan [76, 77]. GPI-1 acts during an early event in glycolysis, upstream of GPD-2 and GPD-3, which are both specifically required for a response to anoxic conditions [78]. While it is intuitive that *gpi*-*1* would be required for the normal dauer response to anoxia, it is puzzling as to how *gpi*-*1* may have an effect on the germ line. There were no other members of the glycolytic pathway identified in the RNAi screen, so it seems unlikely that defects in glycolysis are directly responsible for the germline hyperplasia phenotype or that the RNAi effects of these genes are highly penetrant embryonic lethal.

Few genes identified in the *par-4* and *aak(0)* suppressor screen encode proteins that are involved in regulating growth and metabolism. Three out of 4 genes identified in our *par-4* suppressor screen also suppressed AMPK-dependent germline hyperplasia. This suggests that *par-4*/LKB1 regulates growth and metabolism to establish dauer germline quiescence predominantly in an AMPK-dependent manner. This subset includes *cyc-1* (Cytochrome C), *cco-1* (Cytochrome C Oxidase) and *vha-2* (Vacuolar H ATPase)*.* Both *cyc-1* and *cco-1* regulate lifespan and *vha-2(RNAi)* results in sterile animals with polyploidy and premature oocytes [79–82]. All these genes act in a germline non-autonomous fashion making it even more intriguing to characterize how these genes communicate with the germ line during energy stress.

Although one might intuitively assume that the genes identified in our screens function in a germline autonomous fashion, our analysis with *rrf-1* suggested that several genes (Tables 1, 2 and 3) regulate dauer germline quiescence in a germline non-autonomous manner. This represents a novel implication of the somatic tissues in directing mitotic proliferation of GSCs. Further investigation of the subcellular and intercellular structure within the distal germ line or between the germ cells and gonadal sheath cells will enhance our understanding of how these genes influence the regulation of the germ cell population. Though this is a useful approach there are indeed some caveats to consider. The subset of genes that we identified as germline autonomous should not be over-interpreted, as it has been demonstrated that *rrf-1(pk1417)* mutants are still capable of executing RNAi at least in some somatic cells, although this may be at a reduced level [83]. This suggests that some of the genes that are documented as acting in a germline autonomous manner may have also been reduced in somatic tissue, where their function might also contribute to the regulation of germline quiescence.

## Conclusion

Although our analyses are revealing, they are not comprehensive, mainly due to limitations in our RNAi strategy and the essential nature of the process at hand; namely cell cycle dynamics. Nevertheless, our work has revealed the role of several gene families that could act downstream of LKB1/PAR-4 in an AMPK-dependent or -independent fashion to promote germline stem cell cycle arrest under reduced insulin signalling. The data presented here suggest that the tumour suppressor function of LKB1 that is disrupted in Peutz-Jeghers Syndrome patients may be related to its roles in the regulation of cell polarity, and not uniquely due to its ability to activate AMPK.

While AMPK has been shown to be required for the dauer germline quiescence, a direct role for AMPK in PJS has not been established. This suggests that the maintenance of cell polarity by LKB1 that may be disrupted in PJS patients might not involve AMPK, but rather, it may be more due to its disruption in an entirely independent pathway. Alternatively, it could be the combined effect of disrupting both pathways that contribute to the aetiology of the disease. This would be consistent with the poor results obtained using inhibitors of the TOR pathway in the clinic [84]. In this scenario only the downstream effectors of TOR are attenuated, while the polarity of the cells remains compromised, potentially sensitizing PJS cells to continue proliferating through TOR-independent cues.

To our knowledge LKB1 is the sole causative gene in PJS, although others may contribute epistatically. The genes we identified in our unbiased screening approach indicate that the tumour suppressor function of LKB1 may lie in its ability to control cell polarity, and not exclusively in its ability to modulate TOR signalling. This is largely because our *C. elegans*-based strategy allows us to identify genes that affect this LKB1-mediated process that would otherwise be difficult or impossible to characterize in other models, mainly due to their essential nature. It is possible that any functional compromise of these genes in humans would result in lethality, confounding any such functional characterization and thus the absence of additional genes that contribute to the disease.

Further study of the genes we have identified in these screens will provide additional insight as to how PAR-4/LKB1 signalling blocks tumour growth by regulating cell cycle arrest under energetic stress, while providing additional LKB1 or AMPK downstream kinase substrates that could be useful for the development of new therapies to benefit PJS patients or other cancers that arise due to the loss of LKB1 function.

## Methods

### Strains and maintenance

All *C. elegans* strains were maintained at 15 °C and according to standard protocols [85]. The strains used for the screen include MR155 [*daf-2 (e1370) III; qIs56(lag-2::GFP) V*]*,* MR0671 [*rrf-1(pk1417) I; daf-2(e1370) III*], CB1372 [*daf-7(e1372)III*]*,* MR0674 [*daf-2 (e1370) III; par-4(it57) qIs56(lag-2::GFP) V*], MR0672 [*daf-7(e1372) III; par-4(it57) V*], MR0863 [*rrf-1(pk1417) I; daf-2(e1370) III; par-4(it57) V*]*,* MR0998 [*daf-2(e1370) aak-1(tm1944) III; aak-2(ok523) X; qIs56(lag-2::GFP) V*], MR0480[*daf-7(e1372) III; aak-2(ok523) X*], MR0868 [*rrf-1(pk1417) I; daf-2(e1370) III; aak-2(ok523 X)*], MR1842 [*daf-2(e1370) III; orIs20[unc-119(+)Ppie-1::gfp::moesin*], MR2036 [*daf-2(e1370) III; par-4(it57) V; orIs20[unc-119(+)Ppie1::gfp::moesin*]*,* MR2037 [*daf-2 (e1370) aak-1(tm1944) III; orIs20[unc-119(+)Ppie1::gfp::moesin*]. All the strains that possess single gene mutations were obtained from the *Caenorhabditis* Genetic Centre (CGC) unless mentioned otherwise. Transgenic lines and compound mutants were created in the laboratory using standard molecular genetic approaches.

### RNAi screening

Bacterial clones expressing dsRNA from the RNAi library were grown in LB medium with ampicillin at 37 °C overnight. The bacterial culture was seeded onto 12-well NGM plates containing ampicillin and IPTG. Seeded plates were incubated at room temperature for 24 h to induce dsRNA expression. Meanwhile, a population of the animals was synchronized and resulting L1 s were incubated on the dsRNA containing plates at the restrictive temperature (25 °C) to compromise the function of *daf-2* (to induce dauer) and *par-4*. All the worms carrying a *lag*-*2*::*gfp* transgene were examined for DTC displacement, as a proxy for degree of germline hyperplasia [15].

### Germ cell counts

For whole animal DAPI (4′,6-diamidino-2-phenylindole) staining, *C. elegans* dauer larvae were washed off plates and soaked in Carnoy’s solution (60% ethanol, 30% acetic acid, 10% chloroform) for overnight. Animals were then washed twice in PBST (1 × PBS + 0.1% Tween 20), and stained in 0.1 mg/ml DAPI solution for 30 min. Finally, larvae were washed four times (20 min each) in PBST, and mounted in Vectashield (Vector Laboratories) medium. The germ cell nuclei count was determined per dauer larve based on their position and nuclear morphology. The germ cell nuclei count was performed to validate the identified genes from the RNAi screens. The strains (MR0671, CB1372, MR0672, MR0863, MR0480, MR0868) used for the further characterization of the identified genes, didn’t harbour the *lag-2::GFP* transgene and thus the germ cell quantification was performed to study the RNAi effect on the dauer germline quiescence. Two-tailed *t-test* was performed to calculate the *P* value to compare the germ cell count between different genotypes.

### Immunostaining and microscopy

For extruded dauer gonad staining, gonads were dissected, fixed and stained as described elsewhere [86]. Images were captured using the Leica DMR compound microscope equipped with a Hamamatsu C4742–95 digital camera. Image analysis, computational deconvolution and pseudocolouring were performed using Openlab 3.01 software from Improvision. Images were merged and stacked using Image J.

## Additional file

Additional file 1: **Figure S1.** The organization of the actin cytoskeleton is perturbed in the *cdc-42* compromised dauer germline. *daf-2(e1370)* dauer larvae that express the actin cytoskeleton marker were subjected to *cdc-42*(RNAi) and monitored for change in the actin cytoskeletal network in the germline. The image in the left panel consists of merged, condensed Z stacks. Actin filament organization at the membrane adjacent to the rachis is disrupted at 48 h after shifting to their restrictive temperature (arrowhead). *n* = 10 Scale bar: 4 μm. (PDF 84 kb)

## Abbreviations

AMPK: AMP-activated protein kinase
DTCs: Distal tip cells
ECM: Extracellular matrix
GO: Gene Ontology
GSC: Germline stem cell
PJS: Peutz-Jeghers Syndrome
TSC2: Tuberous Sclerosis 2
WASP: Wiskott-Aldrich Syndrome Protein
