# Supplementary figures and images for: Genome-wide surveys reveal polarity and cytoskeletal regulators mediate LKB1-associated germline stem cell quiescence
Source: BMC Genomics. 2018 Jun 15;19:462. doi: 10.1186/s12864-018-4847-y (PMC6003023; doi:10.1186/s12864-018-4847-y)

*daf-2; cdc-42* (RNAi)

Merged

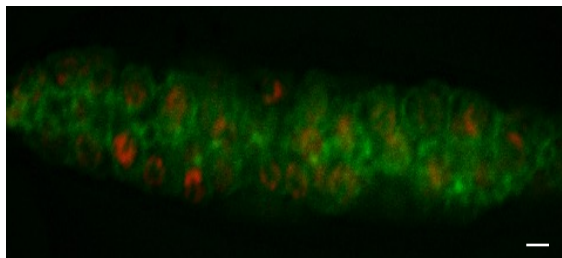

GFP::MOE

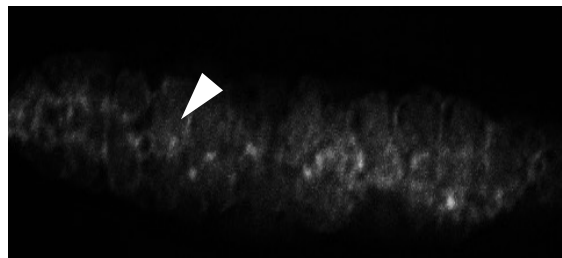

48 hours  
Post L1

Additional File 1

Supplemental Figure 1

Supplement: Supplementary file 1 — Figure S1. The organization of the actin cytoskeleton is perturbed in the cdc-42 compromised dauer germline. daf-2(e1370) dauer larvae that express the actin cytoskeleton marker were subjected to cdc-42(RNAi) and monitored for change in the actin cytoskeletal network in the germline. The image in the left panel consists of merged, condensed Z stacks. Actin filament organization at the membrane adjacent to the rachis is disrupted at 48 h after shifting to their restrictive temperature (arrowhead). n = 10 Scale bar: 4 μm. (PDF 84 kb) [file 12864_2018_4847_MOESM1_ESM.pdf]
